# In Search of a Gold Standard Patient-Reported Outcome Measure for Use in Chemotherapy- Induced Peripheral Neuropathy Clinical Trials

**DOI:** 10.1177/1073274818756608

**Published:** 2018-02-26

**Authors:** Ellen M. Lavoie Smith, Robert Knoerl, James J. Yang, Grace Kanzawa-Lee, Deborah Lee, Celia M. Bridges

**Affiliations:** 1 University of Michigan School of Nursing, Ann Arbor, MI, USA; 2 Phylllis F. Cantor Center for Research in Nursing and Patient Care Services, Dana Farber Cancer Institute, Boston, MA, USA

**Keywords:** chemotherapy-induced peripheral neuropathy (CIPN), QLQ-CIPN20, reliability, validity, sensitivity, responsiveness

## Abstract

**Purpose:**

To test a reduced version—CIPN15—of the European Organisation for Research and Treatment of Cancer (EORTC) Quality of Life Questionnaire Chemotherapy-Induced Peripheral Neuropathy scale (QLQ-CIPN20) to establish a possible gold-standard patient-reported outcome measure for chemotherapy-induced peripheral neuropathy (CIPN).

**Methods:**

Using a prospective, longitudinal, case–control design, patients (n = 121) receiving neurotoxic chemotherapy completed the CIPN15 at baseline and 12 weeks and underwent objective neurological assessment using the 5-item Total Neuropathy Score-Clinical (TNSc). Healthy controls (n = 30) completed the CIPN15 once. Structural validity was evaluated using factor analysis. Because a stable factor structure was not found, a sum score was used to evaluate measures of the CIPN15’s psychometric properties—reliability, validity, sensitivity, and responsiveness—as follows: internal consistency via Cronbach’s α and item–item correlations; test–retest reliability via correlation between 2 CIPN15 scores from each patient; concurrent validity via correlation between CIPN15 and 5-item TNSc scores; contrasting group validity via comparison of CIPN15 scores from patients and healthy controls; sensitivity via descriptive statistics (means, standard deviation, ranges); and responsiveness via Cohen’s *d* effect size.

**Results:**

Most patients received single agent oxaliplatin (33.7%), paclitaxel (21.2%), or more than 1 neurotoxic drug concurrently (29.8%). Factor analysis revealed no stable factor structure. Cronbach’s α for the CIPN15 sum score was 0.91 (confidence interval [CI] = 0.89-0.93). Test–retest reliability was demonstrated based on strong correlations between the 2 scores obtained at the 12-week time point (*r* = 0.86; CI = 0.80-0.90). The CIPN15 and 5-item TNSc items reflecting symptoms (not signs) were moderately correlated (*r* range 0.57-0.72): concurrent validity. Statistically significant differences were found between patient and healthy control CIPN15 mean scores (*P* < .0001): contrasting group validity. All items encompassed the full score range but the CIPN15 linearly converted sum score did not: sensitivity. The CIPN15 was responsive based on a Cohen’s *d* of 0.52 (CI = 0.25-0.79).

**Conclusion:**

The sum-scored CIPN15 is reliable, valid, sensitive, and responsive when used to assess taxane- and platinum-induced CIPN.

## Introduction

Most of the nearly 15.5 million cancer survivors in the United States today received chemotherapy,^1^ a mainstay in cancer treatment. Many chemotherapy drugs are neurotoxic, and almost all individuals who receive these drugs develop some degree of chemotherapy-induced peripheral neuropathy (CIPN).^2^ Common CIPN symptoms—numbness, tingling, and neuropathic pain in the extremities—can necessitate chemotherapy dose reductions, potentially compromising cancer treatment efficacy.^3^ Chronic CIPN often results in diminished functional status and quality of life.^4-6^

Despite the devastating consequences of CIPN, few effective treatments have been identified. Hershman and colleagues rigorously evaluated 48 randomized controlled trials that tested 22 different pharmacologic interventions; of the 48 studies, 47 were negative or inconclusive.^7^ In most of these failed trials, flawed CIPN measurement compromised the ability to uncover effective treatments.^8^ Insensitive and unreliable grading scales, such as the National Cancer Institute’s Common Toxicity Criteria for Adverse Events (NCI CTCAE),^9-13^ were used to measure CIPN outcomes in 22 of the 48 studies.^14-35^ In some cases, investigators not only used weak measures but also used the *wrong* measure altogether: In a recent examination of the research methods of 7 clinical trials testing interventions for painful CIPN, Gewandter and colleagues found that the sole study to uncover an effective treatment (duloxetine)^6^ was the only one using an appropriate pain measure.^36^

The goal of this study was to address the gaps in CIPN measurement science by extensively testing the psychometric properties of a promising CIPN patient-reported outcome (*PRO*) measure, the European Organisation for Research and Treatment of Cancer (EORTC) Quality of Life Questionnaire Chemotherapy-Induced Peripheral Neuropathy scale (QLQ-CIPN20).^37^ Six publications provide empirical evidence supporting the QLQ-CIPN20’s strong internal consistency and stability reliability, sensitivity, validity (content, structural, convergent, concurrent, and contrasting group), and responsiveness.^37-42^ However, 2 studies provide conflicting evidence about the instrument’s structural validity.^39,42^ Another study suggests that the instrument’s factor structure might be improved by item revisions that enhance the instrument’s content validity—the degree to which patients accurately interpret the intended meanings of the items.^38^ Thus, with minor revisions, it has the potential to become the gold-standard PRO measure in CIPN intervention trials.

In this article, we report the results of the last of 3 sequential studies that evaluate the reliability, validity, sensitivity, and responsiveness to change in a revised QLQ-CIPN20 (the modified CIPN15). Results of the first 2 studies, briefly described here, informed this study. The first study (article in press)^43^ was a secondary data analysis to evaluate the sensitivity, internal consistency reliability, and structural validity of the original QLQ-CIPN20 using merged data from European and North American patients (N = 1155) who had participated in studies focused on CIPN measurement, prevention, or treatment.^26,41,44-46^ We found the QLQ-CIPN20 to be sensitive, reliable, and valid; however, items designed to evaluate autonomic neuropathy (dizziness, blurred vision, erectile dysfunction) and hearing loss compromised the instrument’s psychometric properties and were therefore removed. The revised questionnaire was tested for content validity—whether patient understanding of the items was consistent with the intended meanings—in the second study^38^ (N = 25) using established cognitive interviewing techniques.^47^ One additional item (using a car pedal) was removed; several items were retested after rewording to improve clarity. The outcome of these 2 studies was a revised, reduced version—the CIPN15—that was tested in the current study.

## Methods

### Design

We conducted a prospective, longitudinal, case–control study to evaluate the CIPN15’s internal consistency and stability reliability; structural, concurrent, and contrasting group validity; sensitivity; and responsiveness to change.

### Sample and Setting

One hundred twenty patients with cancer from 6 outpatient clinics at the University of Michigan Comprehensive Cancer Center and 30 healthy controls were recruited. The study was approved by the University of Michigan Institutional Review Board (IRB-MED study number HUM00099704). All participants underwent an informed consent process and signed an IRB-approved consent form prior to participating in any study activity.

Patients were eligible if they (1) were to receive neurotoxic chemotherapy for ≥3 months’ duration, (2) had received ≤1/3 of the total planned neurotoxic treatment, (3) were ≥25 years of age, and (4) were willing to complete all study activities. Patients were excluded if they had (1) a <3-month prognosis, (2) peripheral neuropathy from other causes (eg, diabetes, alcohol abuse, and hereditary), and (3) received other nonchemotherapeutic neurotoxic drugs. Healthy controls had neither cancer nor self-reported signs/symptoms of peripheral nerve injury.

### Procedures

Healthy controls completed the CIPN15 only once, immediately following informed consent. The patients receiving neurotoxic chemotherapy (n = 121) completed the CIPN15 at baseline. Twelve weeks later, the patients completed the CIPN15 twice (1-2 hours apart): once before seeing their provider and again prior to receiving chemotherapy premedication. Also, at the 12-week point, patients (not healthy controls) underwent a neuropathy-focused physical examination conducted by 1 of 3 trained nurse examiners using the validated Total Neuropathy Score-Clinical (TNSc).^48-52^ All nurse examiners had undergone neurologist-supervised CIPN assessment training and demonstrated 100% competency on a training checklist and had extensive experience collecting TNSc data. As an incentive to participate, patients received US$10, and healthy controls received US$5.

### Instruments

The original EORTC QLQ-CIPN20, a 20-item self-report questionnaire,^37^ has 3 subscales containing 9, 8, and 3 items assessing sensory, motor, and autonomic CIPN, respectively. Items are scored 1 to 4 with 1 representing “not at all” and 4 “very much.” The total score ranges from 20 to 80 and is converted to a 0 to 100 scale; higher scores indicate worse CIPN. The 15-item version tested in this research, a modification of the original 20-item version, was based on findings from our first 2 studies that are described in one published article^38^ and another in press.^43^ It no longer included the hearing loss, use-of-car-pedals, and 3 autonomic items; 12 of the remaining items had been modified to clarify ambiguous terms or add emphasis.

The extensively validated 5-item TNSc quantifies subjective sensory and motor symptoms, vibration sensation, strength, and reflexes.^4,13,41,48,50,52-59^ Items are rated using a 0 to 4 scale and summed to obtain a total score ranging from 0 to 20. Higher scores reflect more severe neuropathy.

### Data Management

Qualtrics (2017, Provo, Utah, USA), a secure cloud-based service, was used for all data-collection surveys and data storage. Study staff and participants entered all data directly into Qualtrics via tablet computer . Satisfactory mode equivalence between electronic and paper–pencil versions of the QLQ-CIPN20 has been previously demonstrated.^60^

### Analyses

Data were analyzed using R version 3.4.0.^61^ Descriptive statistics (mean and/or frequency, standard deviation [SD], and ranges) were used to describe the sample’s characteristics and CIPN15 and 5-item TNS scores. Structural validity was first assessed using confirmatory factor analysis (CFA) methods. The model’s fit was assessed using the chi-square goodness-of-fit test, comparative fit index (CFI), Tucker–Lewis Index (TLI), and root mean square error of approximation (RMSEA). The RMSEA was the primary measure of fit with values of ≤.05 indicating good fit.^62^ Since CFA did not support a predefined structure, we conducted an exploratory factor analysis using principal axis factoring and oblimin/promax oblique rotation.^63,64^ Bartlett’s test of sphericity and Kaiser-Meyer-Olkin (KMO) measures were used to evaluate item associations. A scree plot guided decisions about the number of factors to explore in the exploratory analysis. Since results from the exploratory factor analysis revealed no clear factor structure, we replicated the methods used by Kieffer and colleagues and evaluated the psychometric properties of the CIPN15 as a mean sum score.^42^ The CIPN15 sum score was calculated using the standard EORTC scoring procedures for symptoms scales:^65^ summing the mean scores for the 15 items and linearly converting them to a 0 to 100 scale, with higher scores indicating worse CIPN.

Given the absence of a strong factor structure, other measures of reliability, validity, sensitivity, and responsiveness were used to evaluate the psychometric properties of the CIPN15 sum score. Internal consistency reliability was assessed using Cronbach’s α and Pearson item–total correlations. Strong internal consistency reliability was indicated by an α ≥ .80 and a correlation range of .30 to .70.^66^ For test–retest reliability, we assessed the Pearson correlation between the 2 CIPN15 scores from the same patient at the 12-week visit. The Pearson correlation between 12-week CIPN15 sum score and 5-item TNS scores was used to assess concurrent validity. Contrasting group validity was assessed by comparing patients’ mean 12-week scores to healthy controls’ mean scores using Welch’s 2-sample *t*-test. Sensitivity of the CIPN15 was determined based on whether patients’ single item scores encompassed the full score range (1-4) and the frequencies of floor/ceiling responses (minimum/maximum scores). Responsiveness to change was assessed using Cohen’s *d* effect size; an effect size of .2 corresponds to a minimal clinically important difference.^67^

There was 89% power to detect medium-sized correlations (*r* = 0.3) and 94% power to detect small–medium differences between CIPN15 mean scores of patients and healthy controls. There was 85% power to detect a medium–large (*d* = 0.65) effect size (indicating responsiveness to change), and 100 participants were adequate for factor analysis.^68,69^

## Results

### Sample Demographic Characteristics

Demographic characteristics of the samples are outlined in Table 1. Patients’ mean age was 57.5 (SD = 11.02, range = 30.0-83.0). Most were female (69.4%) and caucasian (90%). Healthy controls were similar to the patient cohort because they had been matched by age, gender, and race.

**Table 1.** Demographic Characteristics.

Characteristic	Participants Receiving Chemotherapy	Participants not Receiving Chemotherapy
Age		
n	121	30
Mean (SD, Range)	57.5 (11.02, 30.0-83.0)	56.6 (11.37, 34-78)
	n (%)	n (%)
Gender		
Male	37 (30.6)	9 (30)
Female	84 (69.4)	21 (70)
Race		
Caucasian	109 (90)	26 (86.7)
Asian	5 (4.1)	1 (3.3)
Latino/Hispanic	1 (0.08)	1 (3.3)
Multiracial	2 (1.6)	1 (3.3)
African American	2 (1.6)	1 (3.3)
Other	2 (1.6)	0 (0)
Cancer type		
Breast	31 (25.6)	
Colorectal	32 (26.4)	
Pancreas	19 (15.7)	
Multiple myeloma	4 (3.3)	
Lymphoma	6 (4.9)	
Ovarian	12 (9.9)	
Uterine	4 (3.3)	
Vulvar	1 (0.08)	
Other	12 (9.9)	
Neurotoxic drugs received (n = 104)		
Single agents, n (%)	Mean Cumulative Dose Received (n^a^), SD, Range	
Oxaliplatin: 35 (33.7)	947.28 mg (n = 36), 275.51, 365.0-1449.0	
Paclitaxel: 22 (21.2)	1380.80 mg (n = 44), 486.68, 326-2364	
Docetaxel: 0	445 mg (n = 11), 149.93, 154-640	
Vincristine: 5 (4.8)	9.2 mg (n = 5), 1.10, 8.0-10.0	
Cisplatin: 4 (3.8)	406.0 mg (n = 8), 190.58, 193.0-805	
Bortezomib: 3 (2.9)	30.47 mg (n = 3), 11.05, 23.40-43.20	
Abraxane: 2 (1.9)	1053.5 mg (n = 4), 614.27, 440.0-1594.0	
Carboplatin: 1 (1.1)	2517.07 AUC (n = 27), 1153.23, 227-4500	
Vinorelbine: 1 (1.1)	431.0 mg (n = 2), 77.78, 376.0-486.0	
Multiple (administered concurrently or sequentially): 31 (29.8)		
– Cisplatin/paclitaxel (n = 1)		
– Paclitaxel/carboplatin (n = 16)		
– Cisplatin/docetaxel/paclitaxel/carboplatin (n = 1)		
– Docetaxel/carboplatin (n = 6)		
– Cisplatin/vinorelbine (n = 1)		
– Docetaxel/paclitaxel (n = 1)		
– Oxaliplatin/abraxane (n = 1)		
– Abraxane/cisplatin: (n = 1)		
– Paclitaxel/docetaxel/carboplatin (n = 3)		
5-Item TNS at week 12 (n = 104)	Mean (SD), Range	% at Floor^b^
Total score	6.64 (3.17), 0-15	35.6
– Sensory	1.03 (1.06), 0-4	73.1
– Motor	0.51 (0.84), 0-3	89.4
– Strength	0.33 (0.65), 0-3	94.2
– Vibration	2.55 (1.38), 0-4	28.8
– Reflexes	2.23 (1.42), 0-4	34.6

Abbreviations: AUC, area under the curve; SD, standard deviation; TNS, Total Neuropathy Score.

^a^Reflects the number of patients receiving the drug as either a single agent or as part of a multineurotoxic drug regimen.

^b^Total score ≤5 (lowest quartile of total score range), single item scores ≤1.

**Table 2.** Inter-item and Item–Total Correlations.^a^

Item Numbers	1	2	3	4	5	6	7	8	9	10	11	12	13	14	15	Total Score
1	1															0.69
2	0.72	1														0.72
3	0.66	0.48	1													0.76
4	0.44	0.66	0.63	1												0.73
5	0.44	0.40	0.41	0.35	1											0.70
6	0.44	0.53	0.40	0.47	0.68	1										0.68
7	0.18	0.17	0.29	0.26	0.39	0.28	1									0.46
8	0.38	0.37	0.53	0.44	0.47	0.55	0.31	1								0.65
9	0.26	0.41	0.42	0.49	0.39	0.28	0.31	0.34	1							0.65
10	0.26	0.28	0.41	0.35	0.52	0.39	0.35	0.30	0.47	1						0.63
11	0.28	0.22	0.39	0.27	0.36	0.32	0.24	0.34	0.48	0.39	1					0.58
12	0.57	0.44	0.69	0.46	0.51	0.41	0.32	0.49	0.50	0.52	0.68	1				0.81
13	0.33	0.31	0.47	0.41	0.50	0.42	0.23	0.41	0.40	0.60	0.47	0.65	1			0.71
14	0.28	0.34	0.33	0.34	0.34	0.36	0.3	0.39	0.46	0.31	0.59	0.57	0.55	1		0.64
15	0.24	0.33	0.31	0.35	0.70	0.19	0.42	0.31	0.63	0.35	0.33	0.45	0.40	0.63	1	0.59

^a^N = 104. Item numbers represent the questions listed in Table 5.

Most patients had been diagnosed with breast (25.6%) or colorectal (26.4%) cancer and received a variety of neurotoxic chemotherapeutic drugs. All had received cumulative chemotherapy drug doses known to cause peripheral neuropathy as defined in published literature.^70-72^ The majority received single agent oxaliplatin (33.7%), paclitaxel (21.2%), or more than one neurotoxic drug concurrently (29.8%). The 5-item TNSc mean score at the 12-week time point was 6.64 (SD = 3.17, range = 0-15). No 5-item TNSc scores were at the top of the range because few patients had motor neuropathy. More specifically, 89.4% and 94.2% of the motor and strength scores, respectively, were at the floor (scores ≤1; Table 1); this was an expected finding, given that 94.1% of the participants received neurotoxic drugs that cause predominantly sensory neuropathy (eg, bortezomib, platinums, and taxanes).^70-72^

### Structural Validity

When testing the CIPN15, CFA results did not confirm that the data were a good fit with 2 previously described subscale structures (sensory/motor or upper/lower extremity).^37,39^ Based on several fit indices, the data were a poor fit with the sensory/motor structure (χ^2^ = 332.793, *P* < .0001; CFI = 0.719; TLI = 0.668; RMSEA = 0.162)^64^ and with an upper/lower extremity structure (χ^2^ = 348.183, *P* < .0001; CFI = 0.701; TLI = 0.647; RMSEA = 0.167).

Since the CFA results did not support the 2 previously described subscale structures,^37,39^ the next step was to conduct an exploratory factor analysis. Bartlett’s test of sphericity (*k* ^2^ = 217.69, *P* < .0001) and the KMO measure of sampling adequacy (0.83) both indicated that the data were factorable.^64^ Following an iterative process wherein we evaluated CIPN15 item loadings for 2- and 3-factor solutions, no clear factor structure emerged (Table 3). Based on the factor loadings from the 2- and 3-factor solutions, all the numbness, tingling, and pain items (except for painful numbness/tingling in fingers/hands) loaded on factor 2. However, factor 1 of the 2-factor solution, and factors 1 and 3 of the 3-factor solution contained a mix of items that were not conceptually aligned with the sensory/motor or upper/lower extremity latent variables suggested in previously published literature.^37,39^

**Table 3.** CIPN15 2- and 3-Factor Solution Loadings From Rotated Factor-Loading Pattern Matrix.^a^

Item	2-Factor Solution		3-Factor Solution		
	1	2	1	2	3
Tingling fingers/hands		0.93		0.81	
Tingling toes/feet		0.92		1.0	
Numbness (loss of feeling) fingers/hands		0.60	0.59	0.42	
Numbness (loss of feeling) toes/feet		0.62		0.63	
Painful numbness/tingling fingers/hands	0.42				
Painful numbness/tingling toes/feet		0.49		0.48	
Cramps fingers	0.45				0.41
Cramps toes					
Balance	0.63				0.71
Hot/cold water	0.64		0.44		
Holding fork/knife	0.83		0.78		
Small objects	0.71		0.95		
Open jar	0.76		0.63		
Ankle flex weakness	0.81				0.57
Get up out of chair (leg weakness)	0.68				0.90

Abbreviation: CIPN, chemotherapy-induced peripheral neuropathy.

^a^N = 104. Principal axis factoring with promax rotation; loadings < 4.0 are not reported.

### Internal Consistency and Test–Retest Reliability

The CIPN15 sum score exhibited strong internal consistency reliability as evidenced by a Cronbach’s α of .91 (confidence interval [CI] = 0.89-0.93) and moderate to high item-total score correlations (Table 2). Excellent test–retest reliability was demonstrated based on strong correlations between the 2 scores obtained at the 12-week time point (*r* = .86; CI = 0.80-0.90).

### Concurrent and Contrasting Group Validity

Concurrent validity was evidenced by moderately strong positive correlations between the mean 5-item TNSc total score and the CIPN15 sum score (*r* = .57). Further, the 5-item TNSc subjective sensory and motor scores correlated moderately with the CIPN15 sum score (*r* = .57 and .72, respectively; Table 4). However, as expected based on prior research,^40^ the 5-item TNSc preclinical sign scores (ie, vibration and reflexes) did not highly correlate with the CIPN15 sum score reflecting patient-reported symptom severity. Contrasting group validity was demonstrated based on statistically significant differences in CIPN15 sum scores when comparing patient scores (mean = 14.27, SD = 17.33) to control group scores (mean = 0, SD = 0; *P* < .0001). Control group patients had no evidence of CIPN.

**Table 4.** Concurrent Validity Based on 5-Item TNS and CIPN15 Sum Score Correlations.^a^

Subjective Sensory *r* (CI)	Subjective Motor *r* (CI)	Strength *r* (CI)	Vibration *r* (CI)	Reflex *r* (CI)	Total *r* (CI)
0.57 (0.42 to 0.68)	0.72 (0.62 to 0.80)	0.35 (0.17 to 0.51)	0.19 (−0.01 to 0.37)	0.08 (−0.11 to 0.27)	0.57 (0.42 to 0.69)

Abbreviations: CI, 95% confidence interval; CIPN, chemotherapy-induced peripheral neuropathy; *r,* Pearson correlation; TNS, Total Neuropathy Score.

^a^N = 103. All correlations based on 2-tailed test.

### Sensitivity and Responsiveness

**Figure 1.**
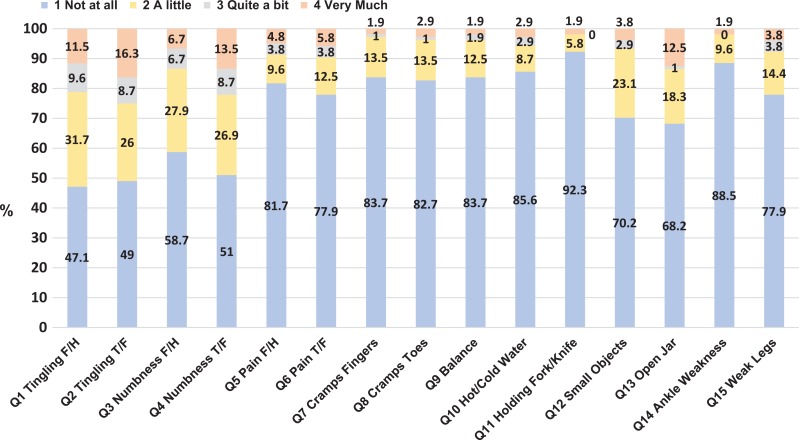
Item response frequency. T/F indicates toes/feet; F/H, fingers/hands.

Table 5 provides item and subscale score descriptive statistics and the frequency of lowest (floor) and highest (ceiling) scores. Figure 1 further illustrates the frequencies of full range 1 to 4 responses for all 15 items. All individual items covered the full 1 to 4 range and thus were sensitive: able to distinguish subtle changes in CIPN.^73^ However, when the CIPN15 sum scores were transformed to a 0 to 100-point scale, no scores reached the ceiling (100), suggesting a floor effect. The effect size for the CIPN15 sum score was small-medium and clinically significant (*d* = 0.52; CI = 0.25-0.79).

**Table 5.** Scoring Ranges for Items and Sum Score.

	Mean	Median	Standard Deviation	Range	% at Floor	% at Ceiling
Items						
Q1-Tingling fingers	1.86	2.0	1.0	1-4	47.1	11.5
Q2-Tingling toes	1.92	2.0	1.11	1-4	49.0	16.3
Q3-Numbness fingers	1.62	1.0	0.88	1-4	58.7	6.7
Q4-Numbness toes	1.85	1.0	1.06	1-4	50.9	13.5
Q5-Painful numbness/tingling fingers	1.32	1.0	0.77	1-4	81.7	4.8
Q6-Painful numbness/tingling toes	1.38	1.0	0.81	1-4	77.9	5.8
Q7-Cramps fingers	1.21	1.0	0.55	1-4	83.7	1.9
Q8-Cramps toes	1.24	1.0	0.62	1-4	82.7	2.9
Q9-Balance	1.22	1.0	0.57	1-4	83.7	1.9
Q10-Hot/cold water	1.23	1.0	0.64	1-4	85.6	2.9
Q11-Holding fork/knife	1.12	1.0	0.47	1-4	92.3	1.9
Q12-Small objects	1.40	1.0	0.73	1-4	70.2	3.8
Q13-Open jar	1.58	1.0	1.01	1-4	68.3	12.5
Q14-Ankle flex weakness	1.15	1.0	0.50	1-4	88.4	1.9
Q15-Get out of chair (Weak legs)	1.34	1.0	0.73	1-4	77.9	3.8
CIPN15 Sum score	14.27	8.89	17.33	0-73.3	21.2	0

Abbreviation: CIPN, chemotherapy-induced peripheral neuropathy.

## Discussion

Which CIPN PRO measure to use and whether a PRO measure will adequately quantify CIPN in the absence of objective assessments using physical examination, nerve conduction study, skin biopsy, and/or quantitative sensory testing techniques are widely debated questions. The strongest argument for using a PRO measure in clinical trials is that PRO measures quantify patients’ experiences. Further, PRO measures are feasible for use in multisite clinical trials and can be used alongside objective measures (eg, skin biopsies, TNS examinations, and quantitative sensory tests) to enhance measurement validity.

Our findings reflect the culmination of 3 sequential National Institutes of Health (NIH)-funded studies designed to test and optimize the QLQ-CIPN-20. Using quantitative (reflected in the current results and another paper in press) and qualitative methods,^38^ we tested the QLQ-CIPN20 via iterative test/revision/retest cycles using samples of patients who had received many different types/classes of neurotoxic chemotherapy. Based on this work, we recommend that the EORTC consider adapting the 20-item PRO measure to a shorter 15-item version that incorporates subtle changes to item wording.^38^ This recommendation is based on strong evidence that the QLQ-CIPN15 sum score is reliable (based on internal consistency and test–retest reliability testing) and valid (based on concurrent and contrasting group validity testing). Individual item scores were sensitive because the scores encompassed the entire 1 to 4 range, but the linearly converted sum score did not encompass the entire score range: There were no participant scores at the ceiling. The 15-item version was also responsive to change. Finally, the shorter version is more parsimonious and thus easier to complete.

Similar to the Kieffer et al approach,^42^ we evaluated the psychometric properties of the CIPN15 sum score. However, this CIPN20 variant was slightly different than what Kieffer’s group tested. Some of the CIPN15 items had been changed slightly to improve content validity based on our published results, and we did not include the autonomic items from the original 20-item instrument that assessed dizziness, blurred vision, erectile dysfunction, and the hearing and car pedal items. Kieffer and colleagues tested an 18-item version of the original instrument; they eliminated 2 items not answerable by all people (difficulty using car pedals, difficulty maintaining an erection) and did not modify any of the questions. The authors concluded that the instrument does not have a stable factor structure and recommended that all items should be summed. The validity of this approach was supported by statistically significant differences in QLQ-CIPN18 mean sum scores in contrasting patient groups (eg, low/high CTCAE scores, with/without oxaliplatin treatment). Like Kieffer, we were unable to confirm previously published factor structures, and we found that the CIPN15 sum score was reliable and valid based on a variety of psychometric tests.

This study has several limitations. The gap between the 2 times the patients completed the CIPN15 at the 12-week time point—before seeing their provider and again prior to receiving chemotherapy premedication—may not have been sufficiently long to ensure that, when completing the questionnaire the second time, they did not remember the answers they had given the first time. Also, although our sample was comprised of patients with diverse diagnoses and participants received cumulative doses of neurotoxic drugs that are known to result in CIPN, most of our sample received taxanes and/or platinums, and these drugs cause primarily sensory, not motor, neuropathy. Motor CIPN is most common in patients receiving vincas, and our sample did not include a representative sample of these patients. This may explain the low scores on items assessing motor neuropathy (ie, ankle flex weakness, cramps, balance, and leg weakness) and is the main reason no sum scores were at the top of the range. Thus, further instrument testing is needed in the vinca-CIPN population.

Moreover, the CIPN15 was not tested in a racially or ethnically diverse population nor did we collect data about participants’ educational backgrounds. Consequently, we cannot speculate about whether the items were easily interpreted by all study participants regardless of race, ethnicity, or educational level. However, it is important to note that the CIPN20 was previously validated in numerous languages (ie, Italian, Spanish, Greek, Dutch, English, German, and French) by Cavaletti and colleagues using a sample from 8 European countries.^41^ Although our sample was not diverse, Cavaletti’s results suggest that the original CIPN20 instrument is valid and reliable when used in a multilingual European population. Therefore, it is reasonable to hypothesize that the CIPN15 will perform similarly.

A critical challenge to moving forward with the revised CIPN15 is that the instrument is owned by the EORTC, whose established psychometric testing policies require testing in multicultural populations. Given the current results, and previously published evidence supporting the CIPN15’s content validity,^38^ we suggest that the EORTC consider our recommended revisions for a 15-item version. If either the 15-item version discussed here or the 18-item version described by Kieffer and colleagues is used in future CIPN trials, a sum score should be used instead of the sensory, motor, and autonomic subscale scores previously recommended by the EORTC.

Which CIPN PRO measure should be used in future intervention trials? There is no perfect answer to the question because there is no perfect measure. Although the published evidence supports use of the functional assessment of cancer therapy/gynecologic oncology group-neurotoxicity (FACT/GOG-Ntx)^74-76^ or the EORTC QLQ-CIPN,^38,39,41^ the literature does not definitively point to one PRO as superior to the other. The decision about which PRO to use should be informed by comparative psychometric trials that, unfortunately, have not yet been conducted. We initially tested the EORTC QLQ-CIPN20^39^ because its items reflect the CIPN experience, and it has been extensively used^26,32,45,46,44^ and tested in United States^39^ and international studies.^37,40,41^ Moreover, more recent evidence now supports the use of the 15-item version^38^ as well as the QLQ-CIPN18.^42^ When there is no consensus regarding which PRO measure to use in clinical trials, scientific progress is thwarted. Based on the currently available evidence, we recommend that scientists and clinicians use the QLQ-CIPN18—for now. However, we suggest further large-scale psychometric testing of the 15-item version, because the evidence provided by the rigorous, iterative, qualitative, and quantitative methods used in our 3-phase study, of which this is the culmination, lends strong support to future use of the CIPN15.

## Conclusion

A gold-standard CIPN PRO is sorely needed as the first step in identifying new treatments for CIPN. Study results suggest that a revised and abbreviated 15-item version of the EORTC QLQ-CIPN is reliable, valid, sensitive, and responsive for use in North American and European populations receiving taxanes or platinums. Further testing in larger and more diverse samples, which could occur via psychometric subaims embedded within intervention studies, and further validation in patients receiving neurotoxic drugs other than taxanes or platinums (eg, vinca alkaloids, bortezomib), is still warranted.
